# Functional insights into the late embryogenesis abundant (LEA) protein family from *Dendrobium officinale* (Orchidaceae) using an *Escherichia coli* system

**DOI:** 10.1038/srep39693

**Published:** 2016-12-22

**Authors:** Hong Ling, Xu Zeng, Shunxing Guo

**Affiliations:** 1 Institute of Medicinal Plant Development, Chinese Academy of Medical Sciences, Beijing, 100193, China

## Abstract

Late embryogenesis abundant (LEA) proteins, a diverse family, accumulate during seed desiccation in the later stages of embryogenesis. LEA proteins are associated with tolerance to abiotic stresses, such as drought, salinity and high or cold temperature. Here, we report the first comprehensive survey of the LEA gene family in *Dendrobium officinale*, an important and widely grown medicinal orchid in China. Based on phylogenetic relationships with the complete set of *Arabidopsis* and *Oryza* LEA proteins, 17 genes encoding *D. officinale* LEAs (*DofLEAs*) were identified and their deduced proteins were classified into seven groups. The motif composition of these deduced proteins was correlated with the gene structure found in each LEA group. Our results reveal the *DofLEA* genes are widely distributed and expressed in tissues. Additionally, 11 genes from different groups were introduced into *Escherichia coli* to assess the functions of *DofLEAs*. Expression of 6 and 7 *DofLEAs* in *E. coli* improved growth performance compared with the control under salt and heat stress, respectively. Based on qPCR data, all of these genes were up-regulated in various tissues following exposure to salt and heat stresses. Our results suggest that *DofLEAs* play an important role in responses to abiotic stress.

In nature, abiotic stresses, such as drought, high temperature, salinity, cold, and heavy metal pollution, have a serious effect on plant growth and development^1^, with drought, high temperature, and salinity being associated with water limitation. High temperature damages cell membrane integrity, inhibits photosynthesis, and leads to cellular aging and death. Salt stress decreases total biomass, limits photosynthesis and affects the metabolic system^1^,^2^,^3^. Higher plants have evolved a wide variety of defense mechanisms in response to adverse conditions, often manifesting in the synthesis of a series of functional proteins to reduce damage and protect cells. For example, accumulation of late embryogenesis abundant (LEA) proteins is an important response to abiotic stress^4^,^5^.

LEA proteins were first shown to accumulate in seeds during the later stages of embryogenesis. Since then, these proteins have been detected in seedlings, stems, and roots and also in anhydrobiotic bacteria and invertebrates^6^. LEA proteins comprise a group of small and hydrophilic polypeptides (MW = 10–30 kDa). They are predominantly composed of a repeating arrangement of hydrophilic amino acids that form a highly hydrophilic structure with high heat stability. In addition, LEA proteins have been shown to protect plant metabolism against abiotic stresses, with properties that include antioxidant activity, metal ion binding, membrane and protein stabilization, hydration buffering, and DNA and RNA interactions^4^,^6^.

Based on repeated motifs, amino acid composition and phylogenetic relationships, plant LEA proteins can be classified into 8 subgroups (LEA1, LEA2, LEA3, LEA4, LEA5, LEA6, dehydrin and SMP), as opposed to 6 or 7 subgroups suggested by prior studies^7^,^8^. However, LEA family proteins have thus far only been investigated in a few plant species, such as *Arabidopsis*, rice, poplar, Chinese plum, legume, tomato, potato, barley and *Pinus*^9^,^10^,^11^,^12^,^13^,^14^,^15^,^16^. In contrast, very few LEA genes have been identified in Orchidaceae, and studies on LEA family evolutionary relationships and functional characteristics are scarce.

*Dendrobium officinale*, a herbaceous perennial plant, has great ornamental and economic value as one of the most important plants used in traditional Chinese medicine. *D. officinale* is a typical epiphytic orchid. It grows compatibly on tree trunks in primeval forests or on damp rock of mountain climates at 500–1600 meters in warm and humid environments^17^. However, this orchid is easily influenced by abiotic stress, such as drought, high temperature, and salinity, causing an extremely low natural reproduction rate and slow growth in the wild^18^,^19^,^20^. Therefore, it is important to understand the mechanisms underlying the response of *D. officinale* to abiotic stress. The recent publication of the *D. officinale* genome has enabled the identification and characterization of the complete repertoire of LEAs in this plant. In the present study, we performed genome-wide analysis of LEA genes in *D. officinale*. We also investigated the characteristics of the LEA gene family in *D. officinale (DofLEA*), including evolutionary relationships, putative functions, expression patterns in various tissues and responses to different abiotic stresses.

## Results

### LEA genes in the *D. officinale* genome

**Figure 1:**
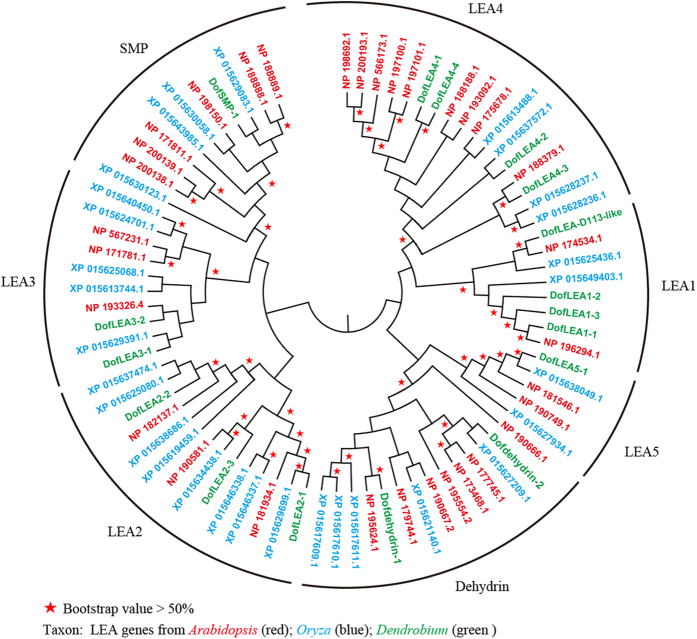
Phylogenetic relationships of *D. officinale, Oryza* and *Arabidopsis* LEA proteins.

Based on the *D. officinale* genome and seed transcriptome, TBLASTN searches using *Arabidopsis* and *Oryza* LEA proteins as query sequences identified 30 different LEA genes (*DofLEA*s). Subsequently, we designed primers and successfully cloned 15 and 2 LEA genes from *D. officinale* seeds and roots, respectively (listed in Supplementary Table S1). Phylogenetic analysis of the predicted proteins revealed that the 17 *DofLEA* genes could be classified into seven groups together with the *Arabidopsis* and *Oryza* LEAs, including 4 LEA1, 3 LEA2, 2 LEA3, 4 LEA4, 1 LEA5, 2 dehydrin and 1 SMP protein-encoding genes (Fig. 1).

Analysis of physicochemical properties (Table 1) revealed that the *DofLEA* proteins have molecular weights ranging from 8.8 to 54.8 kDa, with the smallest proteins belonging to the LEA5 group (~8.8 kDa) and the largest proteins belonging to the LEA2 group (~54.8 kDa). The calculated GRAVY values suggested that all *DofLEA* proteins are quite hydrophilic. Subcellular localization prediction indicated that the LEA1 proteins are located exclusively in the nucleus and mitochondrion; the majority of the LEA2 and SMP proteins are in the cytoplasm, most of the LEA3 proteins in the chloroplast, and the LEA5 and dehydrin proteins in the nucleus.

**Figure 2:**
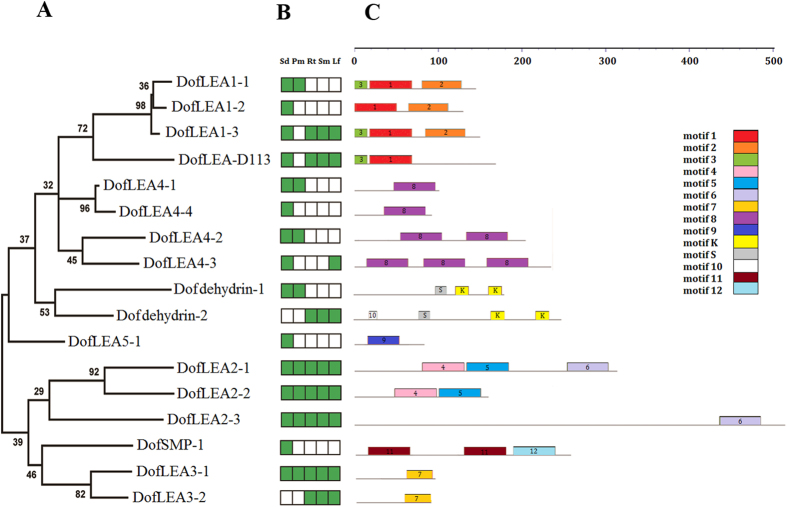
Phylogenetic relationships (**A**), expression patterns (**B**) and motif structure (**C**) of *D. officinale* LEAs. In B, the green box indicates positive detection of gene expression in the corresponding tissue: seed (Sd), protocorm (Pm), root (Rt), stem (Sm) and leaf (Lf). In C, the motif sequences are provided in Supplementary Figure S1.

Motif analysis of the *DofLEA* proteins showed that members of each LEA group possess several group-specific conserved motifs (Figs 2-C and S1). Similar characteristics have also been reported for LEA proteins in *Arabidopsis, Prunus*, poplar and tomato^12^,^13^,^16^,^21^. For example, an important conserved motif in the dehydrin group is the repeated motif, EKKGIMDKIKEKLPG (motif K, richness in lysine residues). In addition, the LEA4 group harbors several repeats of conserved motif 8. The *DofSMP-1* protein contains repeated group-specific motif 11, with two repetitions.

### Expression patterns of *DofLEA* genes in different tissues

We collected five different tissues (seed, protocorm, root, stem and leaf, Fig. 2-B) to analyze the expression pattern of *D. officinale* LEA genes. Based on qPCR results (Figs 2-B and S2), four *DofLEA* genes (*DofLEA2-1, DofLEA2-2, DofLEA2-3* and *DofLEA3-1*) were found to be expressed in all tissues. Four *DofLEA* genes (*DofLEA1-2, DofLEA4-4, DofLEA5-1* and *DofSMP-1*) were only expressed in the seed. In contrast, no detectable seed expression was found for two *DofLEA* genes (*DofLEA3-2* and *Dofdehydrin-2*); thus, we cloned these genes from the *D. officinale* roots. The majority of *DofLEA* genes (15 genes) were expressed in the seed, suggesting that LEA genes play an important role in seed maturation in *D. officinale*. Nine *DofLEA* genes were expressed in vegetative organs (root, stem or leaf), indicating that LEA genes are also involved in normal plant growth and development. Overall, our results showed that the LEA gene family is widely distributed and expressed in this orchid.

### Enhancement of the heat and salt tolerance of transformed *E. coli* (pET28a-*DofLEA*)

To determine the function of LEA proteins in *D. officinale* under stress conditions, we selected 1–2 genes from each group (11 genes in total, shown in Supplementary Figure S3), transformed constructs into *E. coli* (pET28a-*DofLEA*), and applied heat and salt treatments. The cell viability ratio of *E. coli* transformed with the pET28a-*DofLEA* vector and the control containing the empty vector (pET28a) were measured under salt and heat stress.

**Figure 3:**
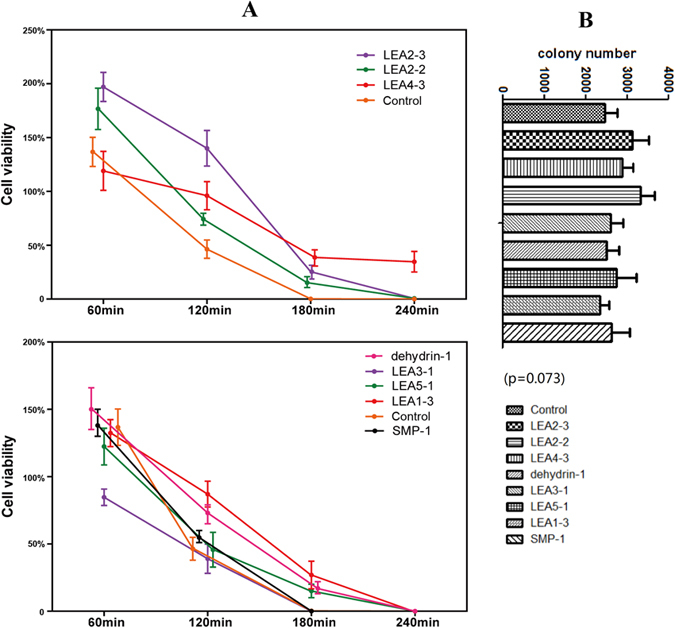
Cell viability ratio of *E. coli* with pET28a-LEA and pET28a (as control gourp) under heat treatment (**A**) and the initial colony number without heat treatment (**B**). (**A**) Cell viability ratio = (colony number on heated plate/colony number on unheated plate) × 100%. The procedure of heat treatment is shown in Figure 6S. The values are the mean ± SE of three samples. (**B**) The initial colony number of un-treated plates (without heat treatment), n = 3, means ± SE (one-way ANOVA, p > 0.05).

In the group exposed to heat treatment, strains carrying 6 *DofLEA* genes (*DofLEA2-2, DofLEA2-3, DofLEA4-3, DofLEA1-3, DofLEA5-1* and *Dofdehydrin-1*) had mean viability ratios 2-10-fold higher than those of the control strain under heat stress (Figs 3 and S4). Interestingly, these *DofLEA* genes belong to different groups. Among these genes, *DofLEA4-3* showed the highest viability ratio after 50 °C induction for 180 and 240 min; after 50 °C induction for 120 min, *DofLEA2-3* had a mean viability ratio ~3-fold higher than that of the control. In addition, the viability ratios for *DofLEA2-2, DofLEA1-3, DofLEA5-1* and *Dofdehydrin-1* were higher than that of the control strain after 50 °C induction for 180 min. There were no differences in mean viability ratios between other LEA genes and the control strain.

**Figure 4:**
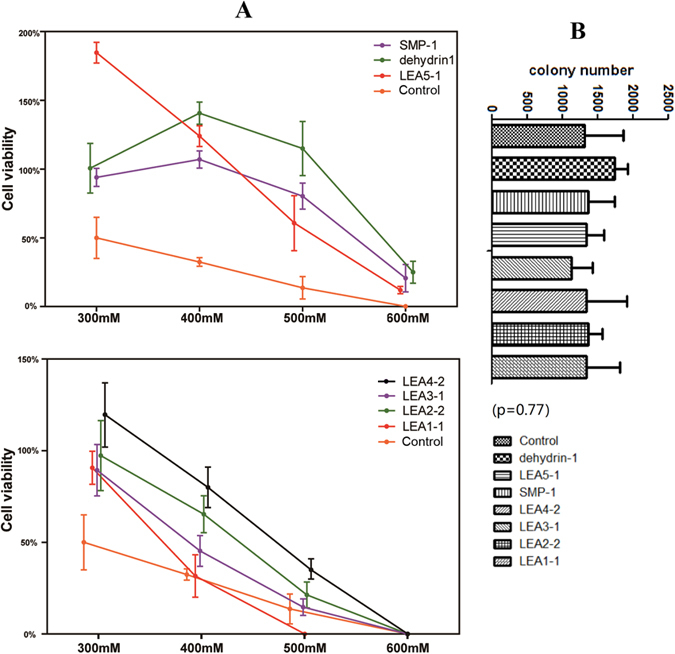
Cell viability ratio of *E. coli* with pET28a-LEA and pET28a (as control group) under salt treatment (**A**) and the initial colony number without salt treatment (**B**). (**A**) Cell viability ratio = (colony number on salted plate/colony number on unsalted plate) × 100%. The procedure of salt treatment is shown in Figure 6S. The values are the mean ± SE of three samples. (**B**) The initial colony number of un-treated plates (without salt treatment), n = 3, means ± SE (one-way ANOVA, p > 0.05).

After salt treatment (Figs 4 and S5), our results demonstrated that strains carrying 7 of the 11 genes showed signs of increased salt tolerance, with viability ratios higher than that of the control strain. In this case, 6 genes (*DofLEA2-2, DofLEA3-1, DofLEA4-2, DofLEA5-1, DofSMP-1* and *Dofdehydrin-1*) from different groups, but not *DofLEA1-1*, resulted in notably increased salt tolerance. Among these genes, *DofSMP-1* (2-5-fold), *DofLEA5-1* (~3.5-fold), and *Dofdehydrin-1* (2-7-fold) had significantly higher survival ratios compared to the other genes under different concentrations of salt.

### Expression patterns of *DofLEA* genes under stress treatments

**Figure 5:**
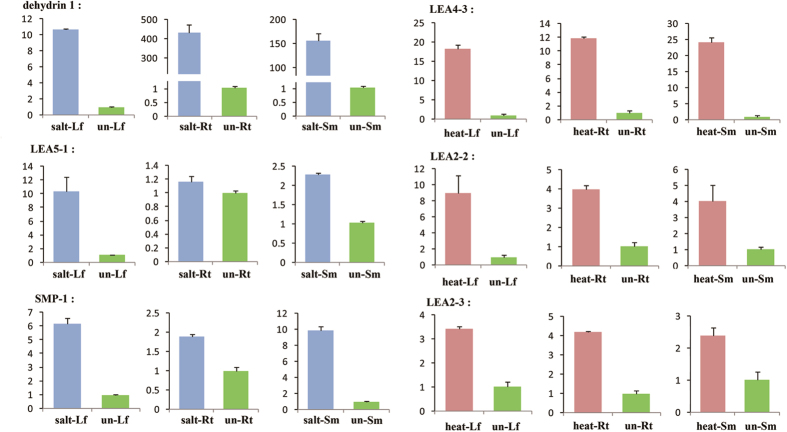
Quantitative PCR analysis of the selected genes. Abbreviations: salt- (salt-treated), heat- (heat-treated), un- (un-treated), Rt (root), Sm (stem) and Lf (leaf), e.g., salt-Lf is the leaf from salt-treated plantlets. Y-axis: relative expression (2^−ΔΔCT^).

To identify *DofLEA* genes with a potential role in abiotic stress response, we investigated the expression patterns of 11 candidate genes using qPCR analysis in *D. officinale* plantlets exposed to heat and salinity. The results of changes in expression of 6 genes are shown in Fig. 5.

Analysis of *DofLEA* gene expression following heat treatment showed induction of *DofLEA4-3, DofLEA2-2* and *DofLEA2-3* mRNA expression in the leaf, stem, and root. Our results also indicated that salt treatment induced the expression of *Dofdehydrin-1, DofLEA5-1* and *DofSMP-1* in the leaf and stem. Overall, the results of expression patterns of most *DofLEAs* under heat or salt stress were consistent with the transformed *E. coli* data, indicating the validity of our experimental results.

## Discussion

Orchidaceae is one of the largest and most widespread families of flowering plants (more than 250,000 species), and the genus *Dendrobium* is one of the largest genera (more than 1000 species). *D. officinale*, an important medicinal herb in China, is critically endangered in the wild^17^. In nature, it grows as an epiphyte on trees or on rocks. Abiotic stress, such as drought, high temperature, and salinity, strongly influences the growth and development of *D. officinale*^18^,^19^. LEA genes have been shown to play important roles in abiotic stress responses of *Arabidopsis, Oryza, Populus* and other plants^11^,^12^,^16^. In contrast, to date, few LEA genes have been described in Orchidaceae. In this study, we identified 30 *DofLEA* genes in the *D. officinale* genome, a number similar to that reported in the genomes of rice (34), *Arabidopsis* (51), *Prunus* (30), poplar (53), tomato (27) and potato (29)^11^,^12^,^13^,^14^,^16^,^21^. In total, we successfully cloned 17 LEA genes from *D. officinale*. Based on phylogenetic analysis, these 17 *DofLEA* genes belong to seven groups of the LEA gene family.

Motif analysis of the proteins encoded by *DofLEA* genes showed that members of each LEA group contain conserved motifs (Fig. 2) that have been previously identified in other plant species, including *Prunus*, potato, tomato and *Arabidopsis*^13^,^14^,^16^,^21^. These results suggest that *DofLEA*s encode functional LEA proteins that have group-specific functions. Additionally, the conserved motifs observed within each LEA group indicate that their members likely originated from gene expansion within the groups.

Our results also indicated that *DofLEA* proteins are rather hydrophilic. Similar characteristics have also been reported for LEA proteins in *Arabidopsis, Prunus*, poplar and tomato, indicating that these proteins are evolutionarily conserved in higher plants^13^,^14^,^16^,^21^.

Subcellular localization analyses showed the present of *DofLEA* proteins in all subcellular compartments, including nucleus, mitochondria, chloroplast, and cytoplasm, as also reported for *Arabidopsis* and tomato LEAs^16^,^21^. There is a strong inference that LEA proteins from the principal groups are ubiquitous within cells and their respective tissues, suggesting that their function is required in all cellular compartments during stress^4^. While our results showed that all the *DofLEA2* were in the cytoplasm. In the case of the maize LEA2 protein, distribution between nucleus and cytoplasm is controlled by phosphorylation of its serine stutter: removal of this sequence results in lack of phosphorylation and retention in the cytoplasm^4^. In addition, our result also showed that two *DofLEA1* proteins were in the nucleus. Prior research classified all of the group 1 LEA proteins as nuclear, and one of these as DNA-binding. Moreover, most of the *DofLEA3* proteins were in the chloroplast. Similarly, CD and FT-IR spectroscopic analyses have revealed that group 3 LEA proteins localize in the cytoplasm, mitochondria, or chloroplasts, and are pre-dominantly unstructured in solution and become mainly α-helical upon desiccation^4^,^22^,^23^.

The development of orthodox seeds concludes by a desiccation phase. The dry seeds then enter a phase of dormancy, also called the afterripening phase, and become competent for germination. At this stage in the development process, seeds acquire the ability to withstand extreme dehydration, LEA proteins have been associated with desiccation tolerance. From our result, 15 *DofLEA* genes were expressed in the seed, suggesting that LEA genes play an important role in seed maturation. Early and middle phases of seed maturation are dominated by the action of abscisic acid (ABA). Subsequently, ABA levels decline and late maturation follows characterised by the synthesis of LEA proteins, associated to the dehydration process and acquisition of desiccation tolerance. Previous studies showed that when ABA was used to activate LEA protein gene expression prematurely in immature soybean seeds, an corresponding improvement in cell integrity was noted after desiccation stress. The plant hormone ABA plays essential roles in several aspects of plant growth and development, including seed maturation, seed dormancy, and adaptation to environmental stresses such as drought and high salinity^4^,^24^,^25^.

Nine *DofLEA* genes were expressed in vegetative organs (root, stem or leaf), indicating that LEA genes are also involved in normal plant growth and development. Among them, three *DofLEA2* and one *DofLEA3* genes were found to be expressed in all tissues. The expression profile of these genes is different from that of the original prototypes (seed-specific or stress-induced expression). Similarly, one dehydrin Xero1 from the group 2 LEA proteins appears to be constitutively expressed. It is not inducible by desiccation or by application of ABA. Prior research has also shown that Q064 31_BETVE, a homologue of the group 3 LEA protein, is itself only constitutively expressed and at low levels^4^.

To date, functional expression screening of LEA proteins from various plant species has been successfully performed in *E. coli* exposed to abiotic stress. Previous study showed that a *Brassica napus* LEA4 gene enhanced the tolerance of *E. coli* to high temperature and salt stresses^26^. Another study showed that an SMP protein from tea could enhance *E. coli* tolerance to various stresses^27^. Liu *et al*.^28^ investigated a maize LEA3 gene expressed in *E. coli* and reported enhanced tolerance to low temperature. Previous studies have primarily focused on the major groups LEA3, LEA4, SMP and dehydrin, but no Orchidaceae LEA genes have been examined. In this study, 6 (~55%) and 7 (~64%) of the *DofLEA*s analyzed increased the tolerance of *E. coli* to heat and salt stresses, respectively. These genes are members of almost all groups of the LEA family, which indicates that these genes have a function in stress response. Thus, we showed for the first time that *D. officinale* LEA proteins from different LEA subgroups enhance tolerance to salt and heat exposure. Our results indicate that LEA proteins play an important role in plant acclimation to stress conditions.

The LEA subgroups represent diverse adaptation to heat and salt stresses. Based on our results, LEA1 increased bacterial resistance to heat and salt, but not significantly; the viability ratio was 1-2-fold higher than that of the control strain. Dehydrin, SMP and LEA5 induced higher tolerance to salt stress (more than 3-fold higher than the control strain), and LEA2 and LEA4 induced higher tolerance to heat (more than 2-fold higher than the control strain), similar with previous studies^26^,^27^. A recent study investigated the functions of six subgroups of LEA proteins from *Arabidopsis* expressed in *Saccharomyces cerevisiae* exposed to desiccation stress, with the results showing that LEA2, LEA4 and dehydrin enhanced tolerance to desiccation stress^29^. While, another functional analysis of three genes from the LEA1, LEA3 and dehydrin groups performed using an *E. coli* expression system supported the hypothesis that LEA1 and LEA3 increased salt tolerance in soybean^30^.

We also used qPCR to investigate the expression patterns of 11 candidate genes in *D. officinale* plantlets under heat and salt stresses. The results indicated that most of the LEA groups tested are expressed in different plant tissues in response to abiotic stresses (heat and salt). Similarly, several LEA1, LEA3, LEA4 and dehydrin genes were also induced by drought stress in *Arabidopsis*, whereas certain LEA3, LEA4, LEA5 and dehydrin genes were induced by salt^16^,^29^,^31^. Additionally, 5 genes from the LEA1, LEA2, LEA4 and dehydrin groups were up-regulated by drought and salt stresses in tomato^21^. In rice, the LEA2, LEA3 and dehydrin groups showed strong responses to osmotic stress, salt and abscisic acid^11^,^32^,^33^.

In conclusion, we for the fisrt time identified and characterized LEA genes in the *D. officinale* genome. The results indicate that LEA proteins are a large family in *D. officinale* and exhibit diverse sequences, motif composition, chromosomal locations and expression patterns. We preliminarily elucidated their functional role and explored their effects on tolerance to abiotic stress (heat and salt).

## Methods

### Plant material and stress treatments

Seeds and tissue-cultured plantlets of *D. officinale* were purchased from Changzhou of Jiangsu. The seed germination procedure for *D. officinale* was described previously^17^. Then, samples of seeds, protocorms, roots, stems and leaves were prepared. The roots, stems and leaves from unstressed plants were used as the control group. For temperature stress, plantlets were heated at 37 °C for 48 h and the heat-treated tissues (roots, stems and leaves) were collected. For salinity stress, plantlets were treated with 0.5 M NaCl for 48 h and the salt-treated tissues (roots, stems and leaves) were then collected.

### Identification of LEA genes in the *D. officinale* genome

The *D. officinale* genome (downloaded from ftp://202.203.187.112/genome/dendrobe/) and the *D. officinale* seed transcriptome (unpublished) were queried using 51 *Arabidopsis* and 36 *Oryza* LEA protein sequences (downloaded from http://www.ncbi.nlm.nih.gov/) with the TBLASTN tool. All the identified candidates were analyzed using the Pfam database (http://pfam.sanger.ac.uk) to confirm LEA conserved domains. Finally, we retrieved 30 *DofLEA* genes, allowing us to design 30 primers for cloning LEA genes from *D. officinale*.

### Molecular cloning of *D. officinale* LEA genes

Total RNA was extracted from *D. officinale* seeds and roots using an RNeasy® Plant Mini Kit (QIAGEN, Germany) according to the manufacturer’s instructions. First strand cDNA was synthesized using a PrimeScript RT reagent PCR Kit (TaKaRa, Japan), and the synthesized cDNAs were used as templates for PCR with specific primers designed using Primer Premier 6.0 (shown in Supplementary Table S2). The parameters of PCR were as follows: 95 °C for 3 min, 30 cycles of 95 °C for 15 s, and 57 °C for 30 s, followed by 72 °C for 1 min. The PCR products were purified using an Agarose Gel DNA Purification Kit (AidLab, China), inserted into the pTOPO vector (AidLab, China), and sequenced in both directions to verify the LEA sequences. Finally, we successfully cloned 15 and 2 *DofLEA* genes from seeds and roots, respectively. Pfam was used to confirm that these 17 sequences possess LEA conserved domains.

### Bioinformatics analysis

The molecular weight (MW) and grand average of hydropathy (GRAVY) of putative proteins were predicted using PROTPARAM (http://web.expasy.org/protparam/). Multiple sequence alignment was performed using Clustalx with default parameters^34^. Subcellular localization of the proteins was predicted by WoLF_PSORT (http://www.genscript.com/psort/wolf_psort.html). Protein conserved motif analysis was conducted using the program MEME/MAST (http://meme-suite.org). Phylogenetic analysis was performed using the maximum likelihood and neighborjoining methods (bootstrap value = 1000) with MEGA 6.0 software^35^.

### Expression analysis of *DofLEA*s in different tissues

Total RNA was isolated from seeds, protocorms, stems, roots and leaves as previously described^36^. Primers designed using Primer Premier 6.0 are shown in Supplementary Table S3. PrimeScript RT reagent Kit (TaKaRa, Japan) was used for reverse transcription. Subsequently, qPCR was performed in a 15 μL reaction mixture containing 7.5 μL 2 × SYBR^®^ Premix Ex Taq^TM^ II (TaKaRa, Japan), 1 μL cDNA template, 0.3 μL each gene-specific primer and 5.9 μL ddH_2_O. We performed three biological replicates and three technical replicates using the LightCycler® 480 II RT-PCR System (Roche, Switzerland) with its relative quantification software ver. 1.5. The parameters of reactions were as follows: 95 °C for 30 s, 40 cycles of 95 °C for 5 s, and 60 °C for 30 s. The 18S rRNA reference gene was used as an internal control.

### Expression of LEA proteins in *E. coli*

We selected 11 *DofLEA* genes representing each group and transformed them into *E. coli*. The transformed *E. coli* (pET28a-*DofLEA*) cell were grown in our lab as described previously^10^,^30^,^37^. Briefly, *DofLEA* genes were inserted into the pET28a vector and then transformed into the *E. coli* host strain BL21 (DE3). The primers are shown in Supplementary Table S4. Recombinant proteins were induced by the addition of isopropyl β-D-thiogalactopyranoside (IPTG) to a final concentration of 0.5 mM, and incubation continued at 37 °C for 4 h. The bacterial cells were harvested by centrifugation, resuspended in PBS buffer, lysed by sonication, and centrifuged at 12,000 rpm for 10 min. The clarified cell extract was analyzed by 12% SDS-PAGE.

### Heat and salt treatments of transformed *E. coli* cells

Heat and salt tolerance assays were performed as described previously^10^,^30^,^37^ (shown in Figure 6S). The transformed *E. coli* (pET28a-*DofLEA*) colonies grown on Luria-Bertani (LB)+ kanamycin plates were used to inoculate starting cultures, which were incubated at 37 °C for 12–16 h. The cultures were diluted 50-fold using fresh LB medium, and incubation continued at 37 °C until the mid-log phase (2–3 h, OD_600_ = 0.6). After IPTG induction, the cultures were diluted and transferred to 50 °C, sampled at 60, 120, 180 and 240 min, and plated onto LB+ kanamycin plates. For the salt treatment, after IPTG induction, LB+ kanamycin plates (containing 171 mM NaCl) were supplemented with 300 mM, 400 mM, 500 mM, and 600 mM NaCl. Each 20 μl sample was plated onto LB+ kanamycin plates and then incubated overnight at 37 °C, followed by counting the number of colonies formed to determine cell viability. The colony number on each plate was recorded using CellProfiler software^38^.

The viability ratio of the transformants under heat and salt conditions was calculated according to the following formula^10^: Cell viability ratio = (colony number on stressed plate/colony number on unstressed plate) × 100%. For all experiments (heat and salt), the means of three experiments were determined from three independent transformants. Here, *E. coli* with the empty vector (pET28a) is the control group.

### Expression analysis of *DofLEA*s under stress treatments

Quantitative real-time RT-PCR (qPCR) was used to measure changes in expression in *DofLEA*s between unstressed plantlets and heat-treated or salt-treated plantlets. Total RNA and RT-PCR products were treated as previously described.

## Additional Information

**How to cite this article**: Ling, H. *et al*. Functional insights into the late embryogenesis abundant (LEA) protein family from *Dendrobium officinale* (Orchidaceae) using an *Escherichia coli* system. *Sci. Rep.* **6**, 39693; doi: 10.1038/srep39693 (2016).

**Publisher's note:** Springer Nature remains neutral with regard to jurisdictional claims in published maps and institutional affiliations.

## Supplementary Material

Supplementary Information

## Figures and Tables

**Table 1:** Characteristics of the putative LEA proteins from *Dendrobium officinale*.

Group	Protein	Protein length(aa)	Molecular weight(kDa)	GRAVY value	Predicted Subcellular localization
LEA1	*DofLEA1-1*	143	15.0	−0.964	Mitochondrial
LEA1	*DofLEA1-2*	128	13.9	−0.995	Mitochondrial
LEA1	*DofLEA1-3*	148	15.7	−0.874	Nuclear
LEA1	*DofLEA-D113*	168	17.3	−0.682	Nuclear
LEA2	*DofLEA2-1*	307	34.3	−0.392	Cytoplasmic
LEA2	*DofLEA2-2*	159	17.6	−0.113	Cytoplasmic
LEA2	*DofLEA2-3*	515	54.8	−0.182	Cytoplasmic
LEA3	*DofLEA3-1*	102	11.1	−0.064	Chloroplast
LEA3	*DofLEA3-2*	93	10.2	−0.431	Chloroplast
LEA4	*DofLEA4-1*	102	10.4	−0.947	Mitochondrial
LEA4	*DofLEA4-2*	207	22.3	−0.988	Chloroplast
LEA4	*DofLEA4-3*	238	26.9	−1.616	Nuclear
LEA4	*DofLEA4-4*	93	9.4	−0.954	Mitochondrial
LEA5	*DofLEA5-1*	83	8.8	−1.229	Nuclear
SMP	*DofSMP-1*	257	27.1	−0.429	Cytoplasmic
dehydrin	*Dofdehydrin-1*	181	18.7	−1.127	Nuclear
dehydrin	*Dofdehydrin-2*	250	27.7	−1.179	Nuclear
