# Supplementary material for: Functional insights into the late embryogenesis abundant (LEA) protein family from Dendrobium officinale (Orchidaceae) using an Escherichia coli system
Source: Sci Rep. 2016 Dec 22;6:39693. doi: 10.1038/srep39693 (PMC5177895; doi:10.1038/srep39693)
Supplement: Supplementary Information [file srep39693-s1.pdf]

Functional insights into the late embryogenesis abundant (LEA) protein family from *Dendrobium officinale* (Orchidaceae) using an *Escherichia coli* system

Hong Ling[+], Xu Zeng[+], Shunxing Guo*

Institute of Medicinal Plant Development, Chinese Academy of Medical Sciences, Beijing, 100193, China

*Corresponding: sxguo2015@163.com
[+]These authors contributed equally to this work.

Supplementary

Table S1. LEA protein sequences cloned from *D. officinale*.
Table S2. Primers used to clone LEA genes from *D. officinale*.
Table S3. Quantitative PCR primers for putative genes.
Table S4. PCR primers with enzyme sites used for LEA genes expression.
Figure S1. Conserved motif sequences in *D. officinale* genes.
Figure S2. Quantitative PCR of LEA genes in different tissues.
Figure S3. SDS-PAGE analysis of 11 selected LEA genes from *D. officinale*. Lane: 1/14: Marker; 2/3: −IPTG / +IPTG pET28a; 4/5: −IPTG / +IPTG dehydrin; 6/7: −IPTG / +IPTG SMP-1; 8/9: −IPTG / +IPTG LEA2-3; 10/11: −IPTG / +IPTG LEA1-3; 12/13: −IPTG / +IPTG LEA4-3; 15/16: −IPTG / +IPTG LEA1-1; 17/18: −IPTG / +IPTG LEA2-1; 19/20: −IPTG / +IPTG LEA4-2; 21/22: −IPTG / +IPTG LEA2-2; 23/24: −IPTG / +IPTG LEA3-1; 25/26: −IPTG / +IPTG LEA5-1.
Figure S4. Growth performance of *E.coli* transformed with pET28a-LEA and pET28a (control) constructs under heat treatment.
Figure S5. Growth performance of *E.coli* transformed with pET28a-LEA and pET28a (control) constructs under salt treatment.
Figure S6. The procedure of salt/heat treatment. A: Cell viability ratio formula. B/C: the procedure of salt/heat treatment.

Table S1. LEA protein sequences cloned from *D. officinale*.

**Protein**	**Sequence**
DofLEA1-1	MEAAKEKVSNISATAKAGLDKTKATAEEKVDKVKTRDPLQKEVAEDRKDQKIHQAELEKQQAYEQNAIHKGEAHHTGLGHQTGLGGHHTGVGQHTGLGGHHTGVGHHTGLDETTGGATGLHSTNAPITGTNKHLPGHHTGTGL*
DofLEA1-2	MDKTKATAEEKIEKMKTRDPLQKEMAEERKEEKIRQAELMKQQAYKENAILKGEAQHLGVGHQTGTGLDGHHNGVVPQTGLGEHHTGVRHHIGLVEPTSGAGGLHRTDGPLAGTETNRTAHETGGGLY*
DofLEA1-3	MEAGKEKVLNIGATAKAGMDKTKATAEEKIERMKTRDPLQKEVAEGRKLEKIHQAELAKQQAYKENAIQKGEGEGEAHHIGAGHQTGLVGHHAGVEHQTGLGGHRTAVGHKTGLDEPTNGGTGFHRTDAPIADTKTIQTVQETGGGLY*
DofLEA-D113	MQVAKEKMKDMTSKAKEKMEVQKVKAEEKAEKKAAGTREEKEIAHEFSKARQAEAKMEYHLERAEHRAETLAHRAAGANHVSSNAALTAGGIGGAFHNGHGQPVGGVPVRPGGGAVDAVRPPGTVDAASTYGGVPARPPGGAVYPPAAGMDNGASTYGAGGGKTNDYI*
DofLEA2-1	MASSDNPEVVEREVKEKKHKDEEKNEEKGGFLDKVKDFIQDIGEKIEEAIGFGKPTADVKGVHIPSINLHKAEIVFDVLVSNPNPIPIPLIDINYLIESDGRKLVSGLIPDAGTIHAHGSETVKIPVVLIYDDIKNTYDDIKPGSIIPYRVKVDLIVDVPIIGRLTIPLEKKGEIPVPYKPDVDVEKIHFEKFSFEETIATLHLKLENKNDFDLALNKLDYEIWLSEMSIGSAELNDSTKFEKNGISKMQIPIAFRPKDFGSALWDMIRGRGTGYSIKGNIDVDTPFGAMKLPITKEGGTTRLKKGEDDDGED*
DofLEA2-2	MSSLMDKAKGFVAEKIAHVKMPEASLKDVSVKHLGRDGARFHGEVSVDNPYSHRLPVCELNYTLKSADRVVASGSVMDPGWIEASGLTKLEVPFKVPYDFLMSLLRDIGRDWDIDYVFDVGLTIDLPIVGNFTIPLSTKGELKLPSITDFFSREKSPES*
DofLEA2-3	MVAVFNKELLSWYLITLKLKETVETNLPKNQREPTASSIPLTLYKSDPEFEPAPTPTPPPPPPAPSLDSPWIISIREKLDQARQYEASSPWATLSIYRVPKSLRDVDETAYAPQIVSIGPYHRGRRRLRDMDRHKWRALHHALTRAGHDVRLYLAAARSLEDRARACYESPLSISGEAFVESLVLDAIFALELFRGVAGEGFPRLGYAPNDPVFAVRGIMHSLQRDMIMLENQLPLFVLDIILALQLGHEPSRSGLVAPLALRFFDPLRPTDEPLSSSASFDPLSDAGSSGALHCVDVFRRSLLLACPKPASRPWIRRRWSHARPVADKRRLQLIHCVTELRDAGIKFRRRKSDRFWDIKFKDGILHIPRLLIHDGTKSLFLNLIAFEQCHLECSNHITSYVIFMDNLINSEVDVGYLHHRGIIEHWLGNDGEVADLFNRLCQEVVFDINNCYLSGLSEQMNCYYNHRWNAWRASLSHKYFNNPWAIISLVAAVVLLLLTSAQTFYGIYAYYKPN*
DofLEA3-1	MARALSNGKVLFAALSDGLVLSGRRFSSAAAAAAAGRVMMEETTVLRKGVMKTGTATADEAAMSWIPDPVTGYYRPANCHRETDPAELRQIFLKNKAYV*
DofLEA3-2	MARAFLFATVCRAFICQKRRLSSAAALVTKGQGRATESAPKKNSDTKVVVASDVQNPSTTAWMPDPVTGYYGPANRQKEIDAAEMRGRLFSRK*
DofLEA4-1	MASNEDKAAYHAGEAKGQTQEKKNELLGKASDTAAGAKEKASDAAAGAKDKASNTAEAAKEHAVEGKEKTGGFLQQATETVKNAAQGTTEAVKNTLGIGEKK*
DofLEA4-2	MAVMLNGSRGLFLKLSYPVRSPSALKAKHILFPLKSTQNYCKASASYHEDDRARQIREKPAEAADRAKEVLKEGSESAKERASNVKDTAEDRAKSAADWAKDATNSASEKARNIGDDARESAESAKEKTKESADSAWEKTKESAESTKEKTKEGLGKAAEVAENVGEKAKETVKGVVGAANDTTQKVKDAVVGKDDGAGDNKVHGKW*
DofLEA4-3	MRAYDDNRKEKADEAKEKLRDYSDSTKAKTNEYKDSAAQKTEEVKDNIKEKAGEYKDSAAQKTEESKEKLKDYKNSAAEKAEEAKKNLQDYKDSIAKNARETKDSNAQKLVDYGESTKEKAEETKDSTAQKLGEYKDSAVEKAIEAKDKTGEKAEQTKDETLEKTKESKDTVMGKTVANARRAMELLTGGRKENKQSDDEHLETTKEFGETKKPAQEELEDAEKSLREKEAVVDFGGN*
DofLEA4-4	MSTGDKAAFHAGEAKGQTQEKTDNLFGKASNAASGAKDKTAETAQAAKDHAVEGKDNAGGFLQQTGEKVKNAAQGTTDAVKNTLGIGEENKGN*
DofLEA5-1	MSNVQERRELNEQAEMTVKPGGKGGKSLDAQENLAEGRSRGGQTRKEQIGTEGYKEMGRKGGLGAKAEETGGERATGTGINID*

Dof_dehydrin-1	MDPLGNKVGHGHIDEYGNPITGHGTTGYGDHHHTGGYGAEHGTGAQQLHGHGTAGHVDQHHTGGYGAV<br>HAVQQHTGGGFGTEHGTGAQQLHGGGATPGVLRRSGSSSSSSSSEDDGLGGRRKKNKGIKDKIKEKLPGGH<br>KDSQNPQQHYDQGVAVGGPTQETEKKGIMDKIKEKLPGSHGH*
Dof_dehydrin-2	MAEEFKDQANLNVDGGEGEVRDRGLFDFVHKKKEEEEEKKPQQQEEVLVSGVEKIHIEDGHKVEDKKKEG<br>EKNHGLLEKLHRSHSSSSSSSSSDEEEEVEGEGGIKQKIKKKKQGLKEKLHGREKTEKEEEKKEPAFVPAAAE<br>ETPAIVVEKVDVFEEAAPPPEAEKKGFLDKIKEKLPGHGKKAAEESGAVPPPPLPVVEVPAEHVKEHEVVEGT<br>EGKEKKGFLGKIIEKLPGYHKNAGEEPEKSPGSH*
DofSMP-1	MSKNEQPRRRSDHLDEPIKYGDVFPVQGDLAGEPIRPRDAALMQTAENKITGKTQKDGAAAVMQSAATFNE<br>RAGLVGHDDFTDDAAIRGVTVTETEAPGCRIVRERVAGQTVVNFAVPTATTADEDVAGEKITMGEALEAAG<br>LSQSDRPVEQSDAAAIQAAEVRAIKMTAPRGVAAAAQSAAEINARIAREEDKITVGDVLNNATAKLAPDKEV<br>TREDAEAVMGAELRNRPDLRTVPGGVADSVTAAARLNEKMSRA*

Table S2. Primers used to clone LEA genes from *D.officinale*

Genes	Primer name	Sequence(5’-3’)
D113	RT-DofD113-F	ATGCAGGTCGCAAAGGAGAAGAT
	RT-DofD113-R	CTAAATATAATCGTTGGTTTTGCCACCG
LEA1-1	RT-DofLEA1-1-F	ATGGAAGCCGCTAAGGAGAAGGT
	RT-DofLEA1-1-R	TCCTACAATCCTGTGCCAGTGTGG
LEA1-2	RT-DofLEA1-2-F	ATGGACAAGACGAAGGCTACCG
	RT-DofLEA1-2-R	TCAGTACAAGCCACCGCCAGTC
LEA1-3	RT-DofLEA1-3-F	ATGGAAGCTGGCAAGGAGAAGG
	RT-DofLEA1-3-R	TTAGTACAATCCACCGCCAGTTTCC
LEA2-1	RT-DofLEA2-1-F	ATGGCGTCATCTGACAACCCAG
	RT-DofLEA2-1-R	TCAATCCTCTCCATCATCATCTTCCC
LEA2-2	RT-DofLEA2-2-F	CAATGTCGAGTCTTATGGACAAAGC
	RT-DofLEA2-2-R	TTAGGATTCTGGGCTCTTCTCCCTC
LEA2-3	RT-DofLEA2-3-F	ATGGTCGCCGTCTTCAACAAGG
	RT-DofLEA2-3-R	TGTAGCTAATTTGGCTTGTAGTAGGCA
LEA3-1	RT-DofLEA3-1-F	ATCTATCGAATGGCTCGCGCTC
	RT-DofLEA3-1-R	TTCTCTCTTAAACATAAGCCTTGTTC
LEA3-2	RT-DofLEA3-2-F	ATGGCTCGAGCGTTTCTTTTTGC
	RT-DofLEA3-2-R	TCATTTGCGGCTGAAGAGCCTG
LEA4-1	RT-DofLEA4-1-F	ATGGCGTCGAATGAGGACAAAG
	RT-DofLEA4-1-R	AGCCCCTATTTCTTTTCACCAATTC
LEA4-2	RT-DofLEA4-2-F	ATGGCTGTCATGCTCAACGGAAG
	RT-DofLEA4-2-R	TCACCACTTGCCATGAACCTTAT
LEA4-3	RT-DofLEA4-3-F	ATGAGGGCGTACGATGATAACAG
	RT-DofLEA4-3-R	TCAATTCCCTCCAAAATCCACCACC
LEA4-4	RT-DofLEA4-4-F	ATGTCGACTGGTGACAAGGCTG
	RT-DofLEA4-4-R	GCTAACTAGTTTCCCTTATTTTCCTCTC
LEA5-1	RT-DofLEA5-1-F	AGCTATGTCTAACGTGCAGGAGAG
	RT-DofLEA5-1-R	GCTAGTCGATATTGATCCCCGTCC
SMP-1	RT-DofSMP-1-F	CAGCCATGAGCAAGAATGAACAACC
	RT-DofSMP-1-R	TTAAGCTCTACTCATTTTCTCGTTGAGC
dehydrin-1	RT-Dofdehydrin-1-F	ATGGATCCGTTGGGTAACAAGGT
	RT-Dofdehydrin-1-R	TTAGTGGCCGTGGCTCCCAG
dehydrin-2	RT-Dofdehydrin-2-F	ATGGCTGAAGAGTTCAAGGATCAGG
	RT-Dofdehydrin-2-R	TCAATGACTACCAGGACTCTTCTCAG

Table S3. Quantitative PCR primers for putative genes.

Genes	Primer name	Sequence(5’-3’)
18S	18S-F	TGGACGTGATGAAGGATGGATGAAC
	18S-R	GCAATTCGCACCACATATCGCATT
D113	Q-DofD113-F	AAGAGATCGCTCACGAGTTCT
	Q-DofD113-R	TGCCCATGCCCGTTATGAA
LEA1-1	Q-DofLEA1-1-F	TGGAAGCCGCTAAGGAGAAGGT
	Q-DofLEA1-1-R	TGCCCGAGTCCTGTGTGATGA
LEA1-2	Q-DofLEA1-2-F	ATGGACAAGACGAAGGCTACCG
	Q-DofLEA1-2-R	CCGACTCCTAAGTGCTGTGCTT
LEA1-3	Q-DofLEA1-3-F	GCTGGCATGGACAAGACGAAGG
	Q-DofLEA1-3-R	GCCTGTCTGGTGTTCGACTCCT
LEA2-1	Q-DofLEA2-1-F	ATTGGCGAGAAGATTGAAGAGG
	Q-DofLEA2-1-R	CAGGAATCAGTCCAGACACAAG
LEA2-2	Q-DofLEA2-2-F	CGTTTCCGTCAAGCACCTCG
	Q-DofLEA2-2-R	CGTCAATCCACTCGCCTCAATC
LEA2-3	Q-DofLEA2-3-F	ACTCAGAGGTGGATGTCGGGTA
	Q-DofLEA2-3-R	AGCAGAAGCAGAACAACAGCAG
LEA3-1	Q-DofLEA3-1-F	AAGGTTCTCTTCGCCGCCCTCT
	Q-DofLEA3-1-R	TCCAAGACATCGCCGCCTCATC
LEA3-2	Q-DofLEA3-2-F	AGTATGCCGAGCGTTCAT
	Q-DofLEA3-2-R	ATCCATGCCGTCGTAGAG
LEA4-1	Q-DofLEA4-1-F	TCGGAAAGGCGTCGGACACA
	Q-DofLEA4-1-R	CGCAGCATTCTTCACCGTCTCA
LEA4-2	Q-DofLEA4-2-F	GTTCGCTCTCCTTCAGCACTCA
	Q-DofLEA4-2-R	GCCCTATCCTCTGCCGTATCCT
LEA4-3	Q-DofLEA4-3-F	CAAGGACAGTGCCGTGGAGAAG
	Q-DofLEA4-3-R	CCTTCCGCCCGTCAATAACTCC
LEA4-4	Q-DofLEA4-4-F	CGGCTTCTGGAGCTAAGGACAA
	Q-DofLEA4-4-R	TCTTCACAGCGTCGGTAGTTCC
LEA5-1	Q-DofLEA5-1-F	GCAGGAGAGGCGTGAACTCAAC
	Q-DofLEA5-1-R	CCCACCCTTTCGACCCATCTCT
SMP-1	Q-DofSMP-1-F	GCAGACCGCCGAGAACAAGAT
	Q-DofSMP-1-R	GCAACATCCTCATCCGCCGTAG
dehydrin-1	Q-Dofdehydrin-1-F	GCCGTCCATGCTGTCCAACAA
	Q-Dofdehydrin-1-R	TCCCTCCCAGTCCATCGTCTTC
dehydrin-2	Q-Dofdehydrin-2-F	AGCAGGAGGAGGTTCTGGTCAG
	Q-Dofdehydrin-2-R	GCTGGTACAAACGCAGGCTCTT

Table S4. PCR primers with enzyme sites used for LEA genes expression.

Genes	Primer name	Sequence(5'-3')
LEA1-1	ES-DofLEA1-1-F	***CATATG***GAAGCCGCTAAGGAGA
	ES-DofLEA1-1-R	***GAATTC***CTACAATCCTGTGCCAGTGT
LEA1-3	ES-DofLEA1-3-F	***CATATG***GAAGCTGGCAAGGAGAA
	ES-DofLEA1-3-R	***GAATTC***TTAGTACAATCCACCGCCAG
LEA2-1	ES-DofLEA2-1-F	***CATATG***GCGTCATCTGACAACC
	ES-DofLEA2-1-R	***GAATTC***TCAATCCTCTCCATCATCATCT
LEA2-2	ES-DofLEA2-2-F	***CATATG***TCGAGTCTTATGGACAAAGC
	ES-DofLEA2-2-R	***GAATTC***TTAGGATTCTGGGCTCTTCTCC
LEA2-3	ES-DofLEA2-3-F	***CATATG***GTCGCCGTCTTCAACA
	ES-DofLEA2-3-R	***GAATTC***CTAATTTGGCTTGTAGTAGGCAT
LEA3-1	ES-DofLEA3-1-F	***CATATG***ATCTATCGAATGGCTCG
	ES-DofLEA3-1-R	***GAATTC***TTAAACATAAGCCTTGTTCTTGAGAAATATC
LEA4-2	ES-DofLEA4-2-F	***CATATG***GCTGTCATGCTCAACG
	ES-DofLEA4-2-R	***GAATTC***TCACCACTTGCCATGAACCT
LEA4-3	ES-DofLEA4-3-F	***CATATG***AGGGCGTACGATGATAACAG
	ES-DofLEA4-3-R	***GAATTC***TCAATTCCCTCCAAAATCCACC
LEA5-1	ES-DofLEA5-1-F	***CATATG***TCTAACGTGCAGGAGAGGC
	ES-DofLEA5-1-R	***GAATTC***CTAGTCGATATTGATCCCCGTC
SMP-1	ES-DofSMP-1-F	***CATATG***AGCAAGAATGAACAACCCC
	ES-DofSMP-1-R	***GAATTC***TTAAGCTCTACTCATTTTCTCGTTG
dehydrin-1	ES-Dofdehydrin-1-F	***CATATG***GATCCGTTGGGTAACAAGG
	ES-Dofdehydrin-1-R	***GAATTC***TTAGTGGCCGTGGCTCC

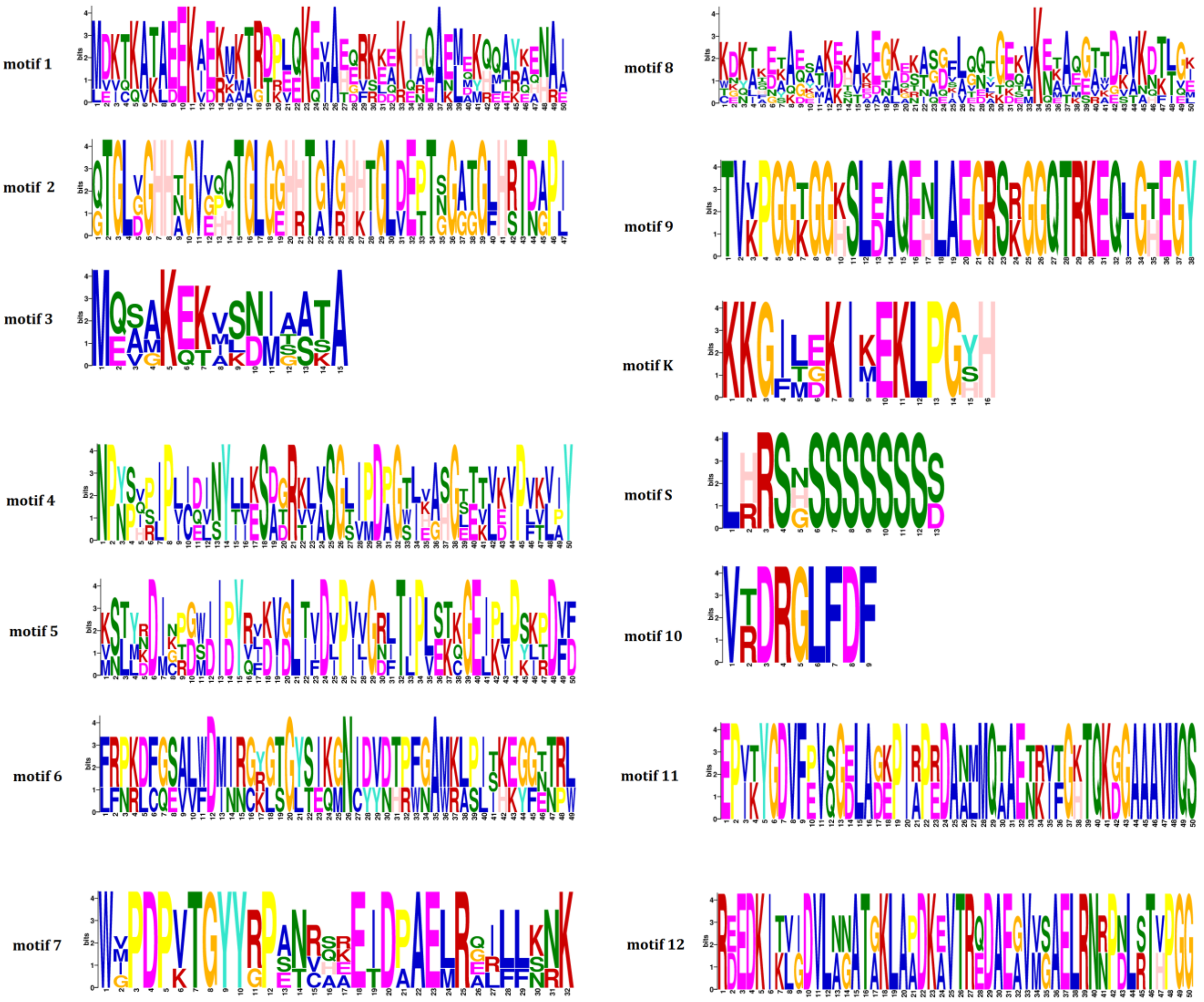

Figure S1. Conserved motif sequences in *D. officinale* genes.

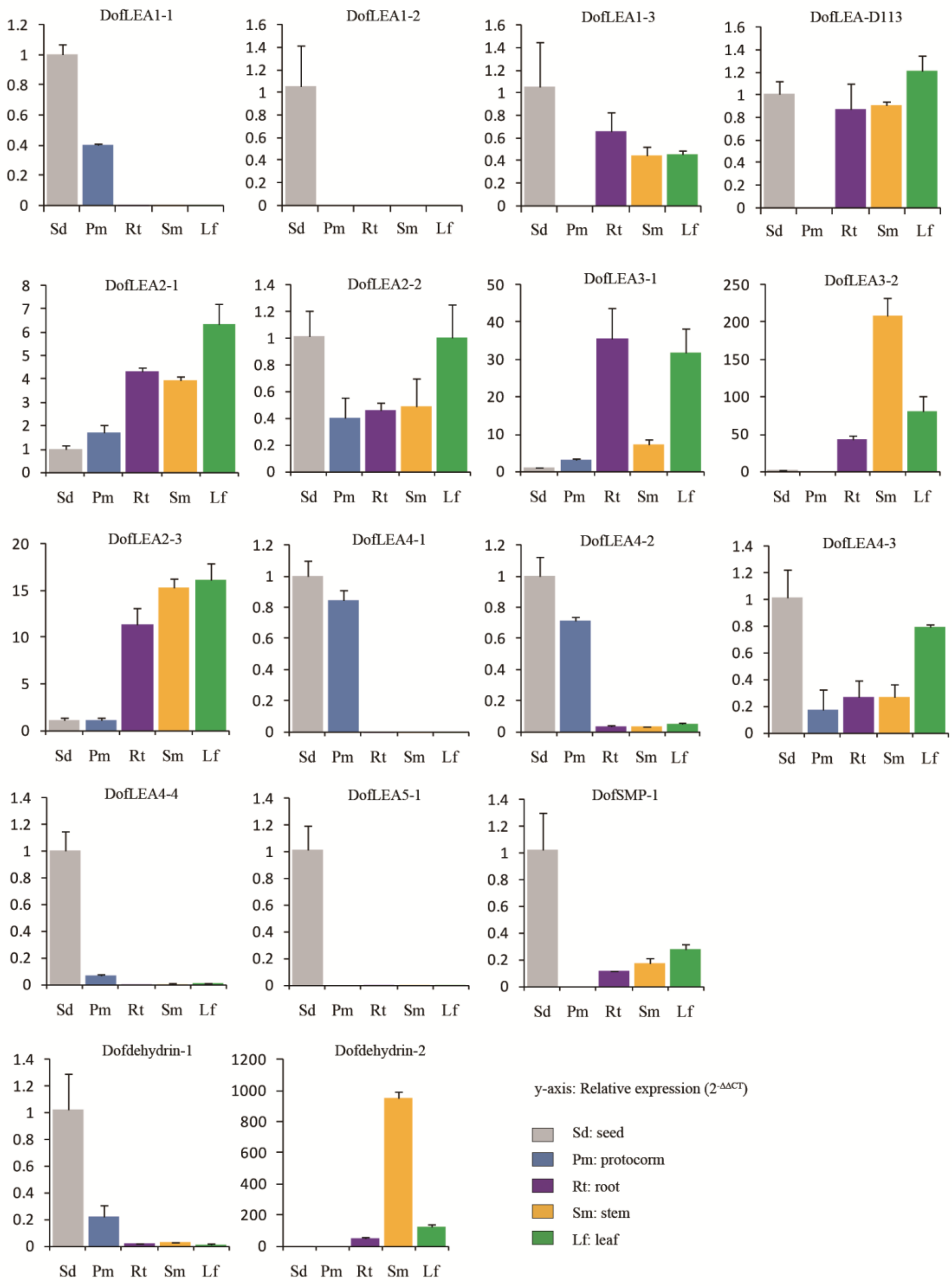

Figure S2. Quantitative PCR of LEA genes in different tissues.

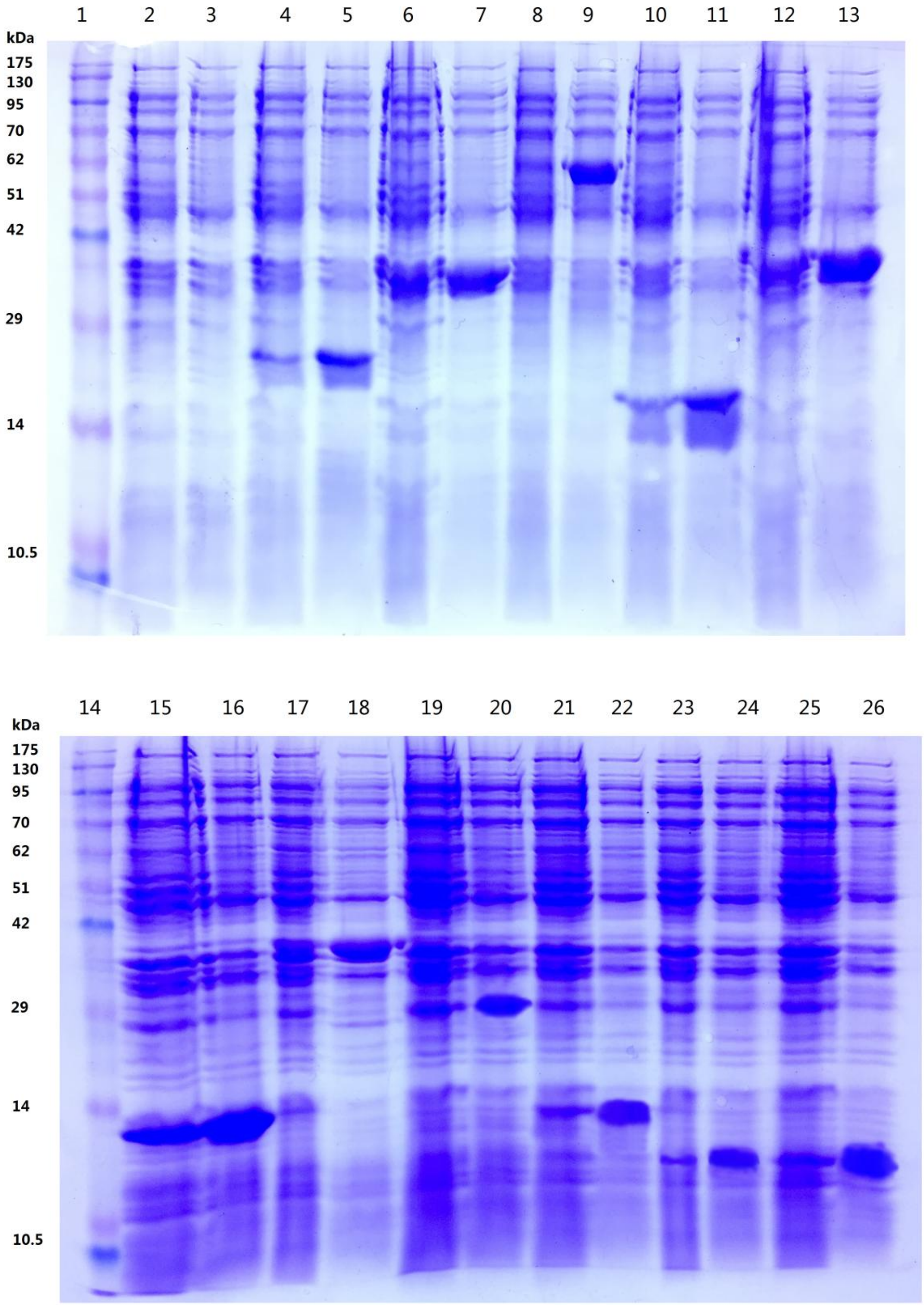

Figure S3. SDS-PAGE analysis of 11 selected LEA genes from *D. officinale*. Lane: 1/14: Marker; 2/3: −IPTG / +IPTG pET28a; 4/5: −IPTG / +IPTG dehydrin; 6/7: −IPTG / +IPTG SMP-1; 8/9: −IPTG / +IPTG LEA2-3; 10/11: −IPTG / +IPTG LEA1-3; 12/13: −IPTG / +IPTG LEA4-3; 15/16: −IPTG / +IPTG LEA1-1; 17/18: −IPTG / +IPTG LEA2-1; 19/20: −IPTG / +IPTG LEA4-2; 21/22: −IPTG / +IPTG LEA2-2; 23/24: −IPTG / +IPTG LEA3-1; 25/26: −IPTG / +IPTG LEA5-1.

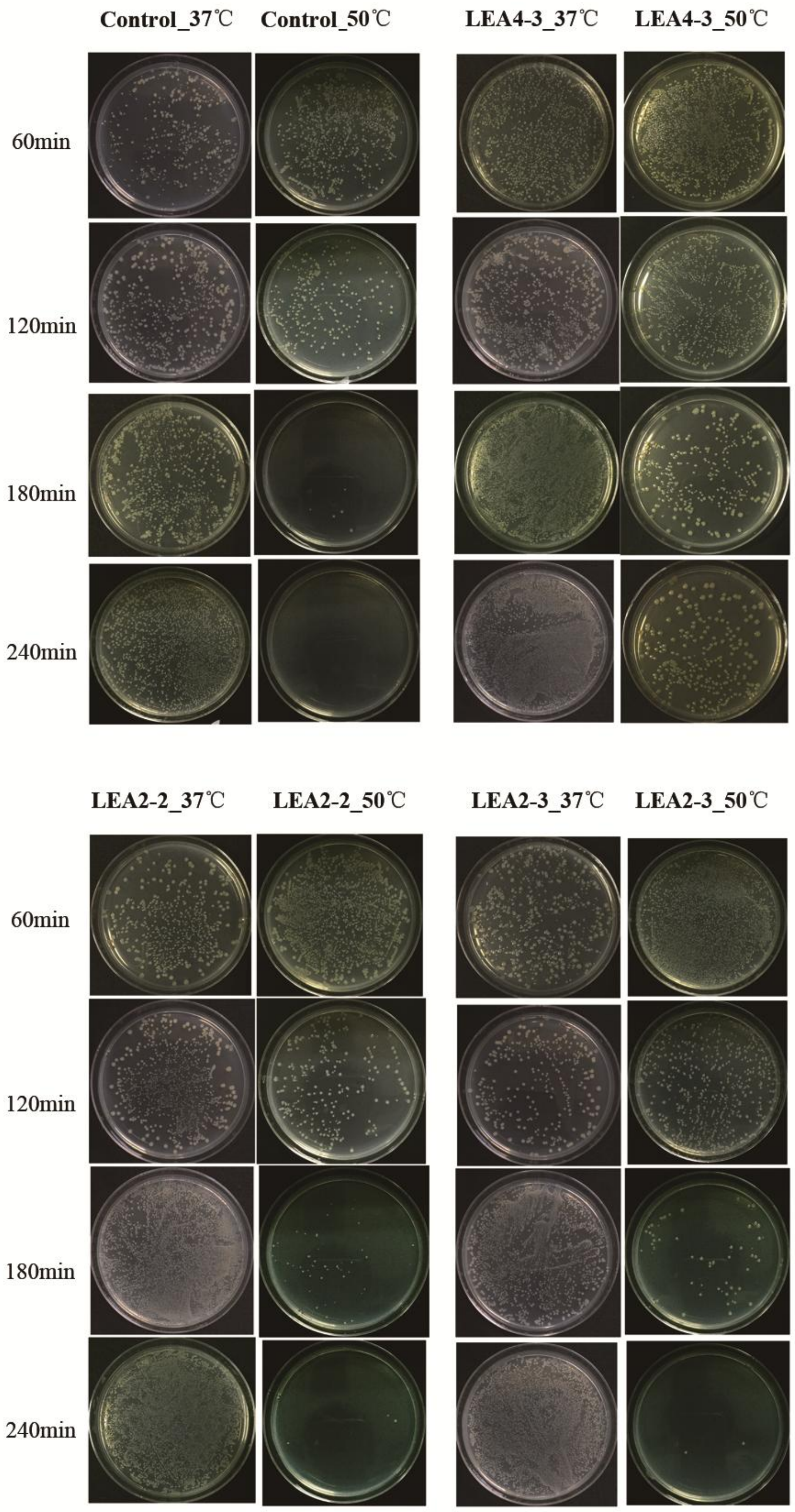

Figure S4. Growth performance of *E.coli* transformed with pET28a-LEA and pET28a (control) constructs under heat treatment.

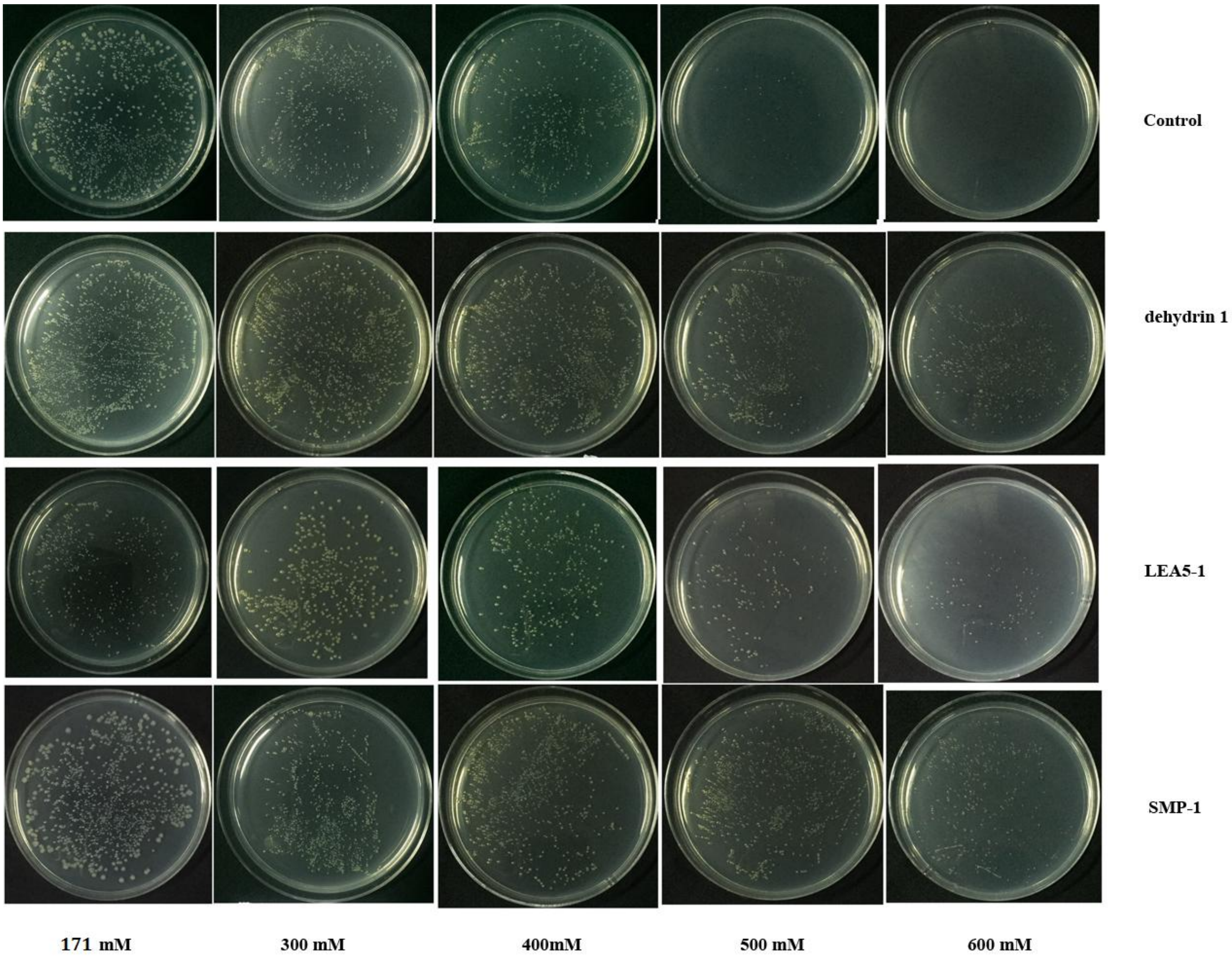

Figure S5. Growth performance of *E.coli* transformed with pET28a-LEA and pET28a (control) constructs under salt treatment.

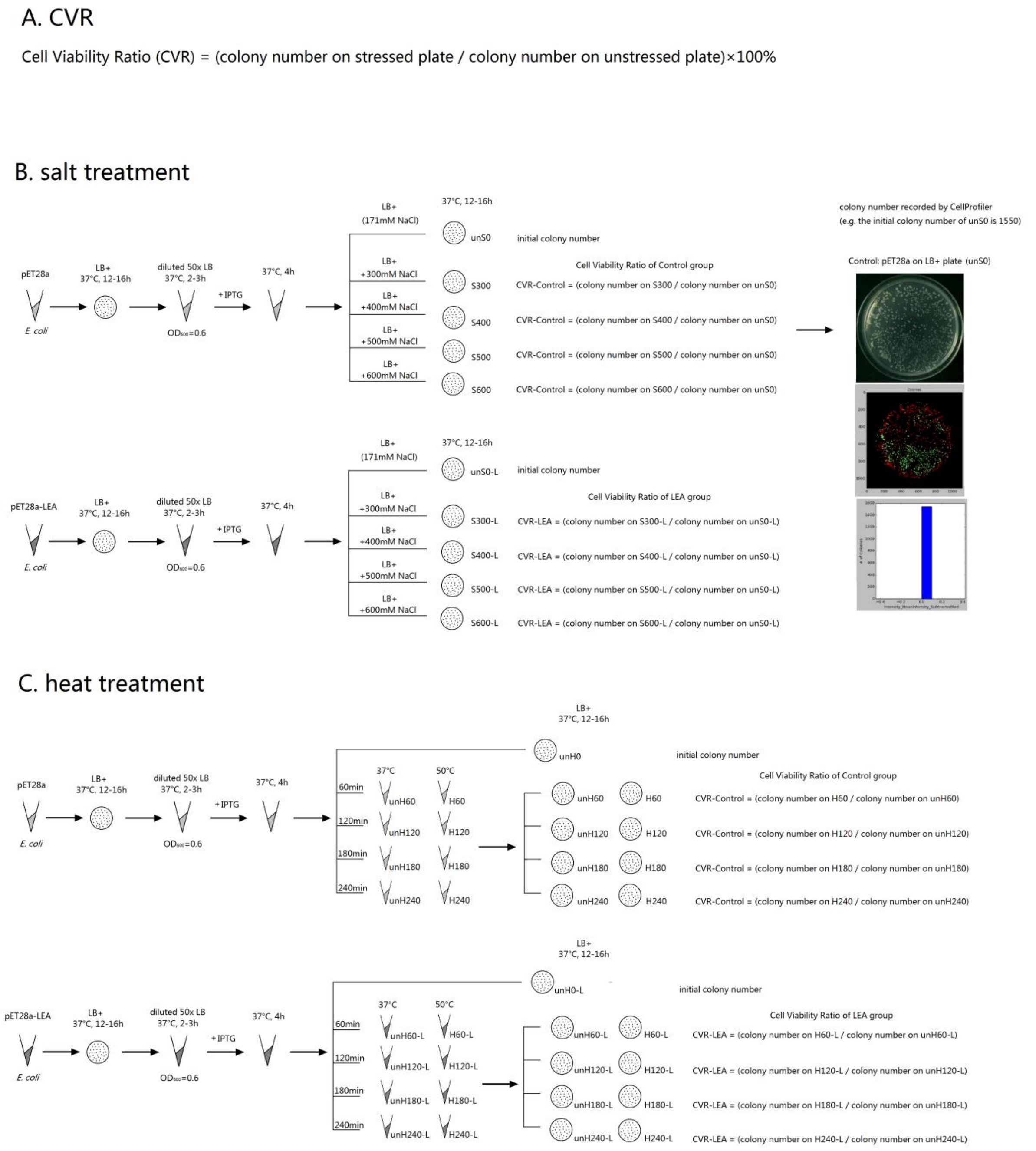

Figure S6. The procedure of salt/heat treatment. A: Cell viability ratio formula. B/C: the procedure of salt/heat treatment.
